# Enhancing patient care quality and safety under the hospital-wide “One Bed” model: a FMEA-based approach

**DOI:** 10.3389/fmed.2026.1681431

**Published:** 2026-02-26

**Authors:** Xixi Li, Yanfei Ma, Haili Long, Jianying Shang, Zhi Zeng, Haiyan Wang, Li Wan, Huaping Huang

**Affiliations:** 1 Department of Nursing, Mianyang Central Hospital, School of Medicine, University of Electronic Science and Technology of China, Mianyang, Sichuan, China; 2 Department of Urology, Mianyang Central Hospital, School of Medicine, University of Electronic Science and Technology of China, Mianyang, Sichuan, China; 3 Department of General Surgery, Mianyang Central Hospital, School of Medicine, University of Electronic Science and Technology of China, Mianyang, Sichuan, China; 4 Intensive Care Unit, Mianyang Central Hospital, School of Medicine, University of Electronic Science and Technology of China, Mianyang, Sichuan, China; 5 Outpatient Department, Mianyang Central Hospital, School of Medicine, University of Electronic Science and Technology of China, Mianyang, Sichuan, China

**Keywords:** Failure Mode and Effects Analysis, nursing, nursing management, nursing quality, One Bed

## Abstract

**Background:**

The hospital-wide “One Bed” model poses potential risks in cross-departmental patient care, affecting nursing quality and patient safety. However, systematic evaluation and improvement strategies for these risks remain limited.

**Objective:**

This study aimed to apply Failure Mode and Effects Analysis (FMEA) to identify and improve nursing quality risks under the “One Bed” model, providing actionable strategies for proactive risk management in cross-departmental patient care.

**Methods:**

Failure Mode and Effects Analysis was employed to systematically identify potential failure modes in nursing processes and quantify associated risks using Risk Priority Numbers (RPNs). Based on RPN values, priority improvement projects were implemented to optimize nursing quality and patient safety.

**Results:**

Following the interventions, RPN values of 15 identified failure modes across pre-, intra-, and post-cross-departmental management all decreased, with reductions ranging from 39.71% to 80.42%, indicating observed improvements in nursing quality and patient safety.

**Conclusion:**

Integrating FMEA into hospital-wide nursing management under the “One Bed” model provides a structured approach for proactive risk identification and workflow optimization. This study offers a practical framework for other institutions seeking to enhance cross-departmental patient care.

## 1 Introduction

Bed management is a critical component of hospital resource allocation and plays a central role in ensuring patient safety and healthcare efficiency. However, existing studies primarily focus on macro-level indicators such as bed turnover and occupancy rates, while giving limited attention to the systematic assessment of nursing quality and safety risks for patients admitted across departments (1, 2). Under cross-departmental admission, patients may receive care in a ward that does not match their primary disease specialty, which can lead to fragmented information transfer, inconsistencies in care processes, and mismatches in nursing competency. These factors may increase the likelihood of delayed clinical decision-making, communication failures, nursing errors, and other preventable adverse events (3–5). Moreover, although patient care quality and safety are core dimensions of healthcare performance, their potential risks under a unified bed allocation model have not been adequately examined.

The hospital-wide “One Bed” model restructures bed allocation by breaking down departmental boundaries and enabling real-time, dynamic redistribution of beds. Since its first implementation at the Sir Run Run Shaw Hospital of Zhejiang University School of Medicine in 1994, this model has gained increasing national attention and has demonstrated advantages in improving bed utilization, admission success rates, and overall operational efficiency. Alongside rising expectations for high-quality care and patient experience, national policies–such as the Notice on Activities to Improve Patient Experience and Enhance Medical Services–have further encouraged hospitals to adopt centralized and unified bed management.

Due to variations in ward expertise and clinical competency, cross-departmental patients are more vulnerable to inadequate monitoring, delayed clinical assessment, and suboptimal delivery of specialized interventions, all of which may compromise nursing quality and safety (6). If these risks are not proactively identified and managed, they may lead to increased safety incidents, heavier nursing workload, disrupted continuity of care, and potentially offset the operational gains achieved by the model (7, 8). Consequently, ensuring the quality and safety of nursing care has become a key challenge in the implementation of the “One Bed” model (9).

The Joint Commission International (JCI) recommends the use of Failure Mode and Effects Analysis (FMEA) as a proactive risk management method to identify potential process failures and mitigate them before adverse events occur (10). FMEA allows systematic decomposition of complex cross-departmental care processes, quantifies potential failure modes, and provides evidence-based guidance for workflow optimization, making it well suited for evaluating nursing risks under the “One Bed” model. Therefore, this study applied FMEA to identify nursing quality risks during cross-departmental hospitalization, develop targeted improvement strategies, and examine whether structured risk management can enhance patient safety and nursing quality within this unified bed management environment.

## 2 Methods

### 2.1 Research subjects

Mianyang Central Hospital is a tertiary comprehensive hospital in Mianyang, with 2,200 open beds. The hospital provides annual outpatient (emergency) services to over 2.336 million visits, inpatient services to over 104,000 patients, and performs over 44,000 surgeries annually, with an average length of stay of 7.1 days.

### 2.2 FMEA implementation process

#### 2.2.1 Formation of the FMEA team

The FMEA team was formed with the participation of hospital leadership and led by the Director of the Nursing Department. It included department heads from 12 functional areas: the Medical Affairs Department, Nursing Department, Security Department, Logistics Department, Infection Control Department, Supply Chain Department, Information Technology Department, and Social Work Department. Team members were well-versed in nursing quality control standards and policies, with a strong awareness of nursing quality risk prevention and quality management. All team members received systematic training in FMEA knowledge and reached a consensus on the team’s objectives, goals, and evaluation criteria.

#### 2.2.2 Process mapping

**FIGURE 1:**
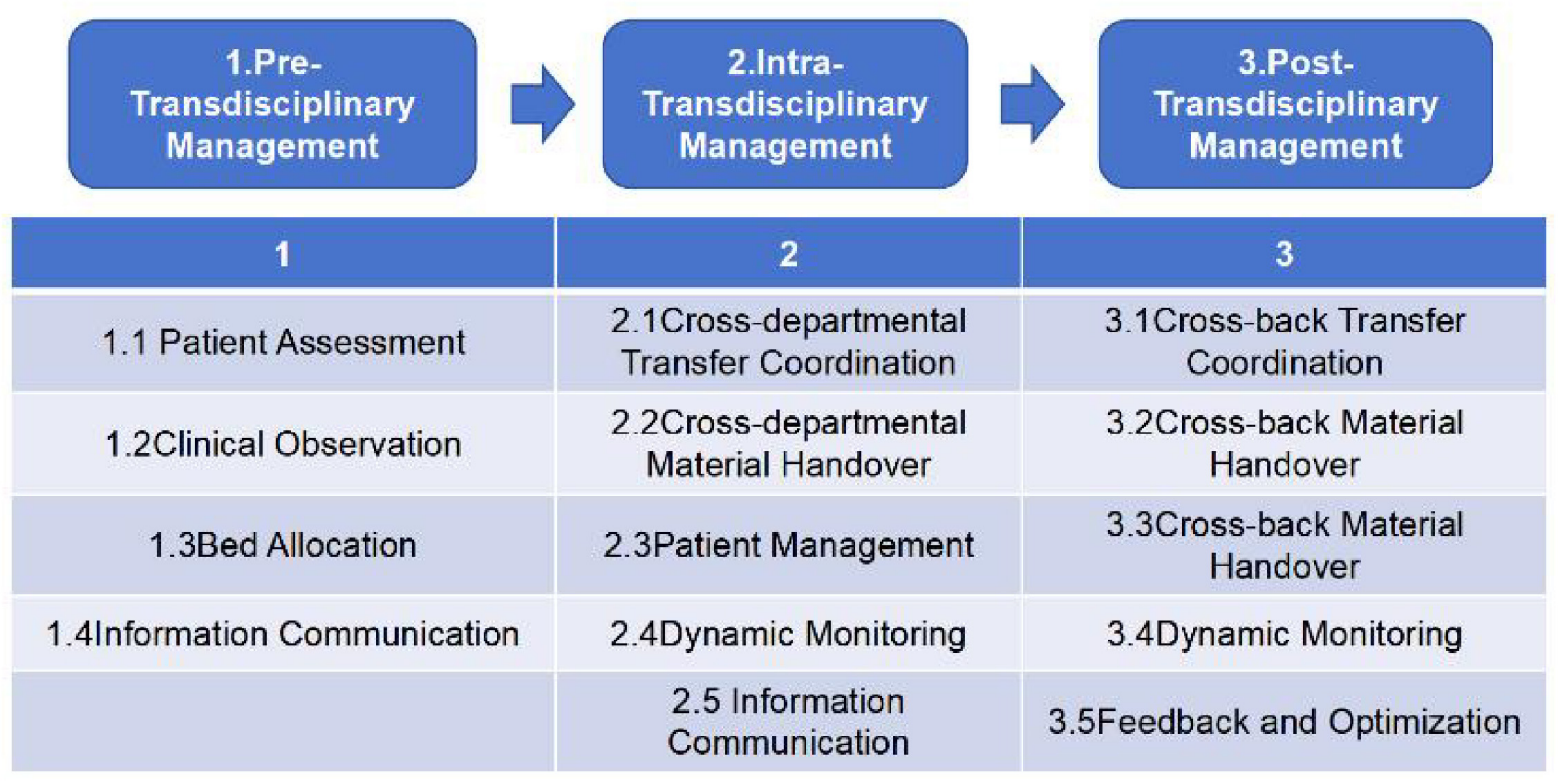
Cross-departmental patient admission process under the “One Bed” hospital-wide model.

Based on the research topic and after discussion by the FMEA team, the patient admission process for cross-departmental cases under the hospital-wide “One Bed” model was defined into three main processes: Pre-Transdisciplinary Management, Intra-Transdisciplinary Management, Post-Transdisciplinary Management. The specific process flow is illustrated in Figure 1.

#### 2.2.3 Identification of potential failure modes

The FMEA team members, based on previous adverse event reports, department interviews, and field investigation results, utilized literature review and brainstorming techniques to identify risk points in the process that are prone to failure. These potential failure points were then compiled into a risk point summary table. The team discussed and outlined the subsystem functional requirements, potential failure modes, possible failure causes/mechanisms, and potential consequences of each step in the process.

#### 2.2.4 Risk assessment

The FMEA team members evaluated each identified risk point, considering the frequency of occurrence (O), the severity of the consequences (S), and the likelihood of detection (D). Severity refers to the potential consequences of a failure, rated on a scale of 1–10, where 1 represents minimal severity and 10 represents extreme severity. Frequency refers to the likelihood of occurrence, rated from 1 to 10, where 1 represents very unlikely and 10 represents highly likely. Detection difficulty refers to the likelihood of detecting the failure after it has occurred, rated from 1 to 10, where 1 indicates it is very likely to be detected in a timely manner, and 10 indicates it is very unlikely to be detected in a timely manner.

This study employed a multi-round Delphi scoring method. In the first round, twelve members of the FMEA team independently evaluated the O, S, and D parameters for each risk point. The project leader then calculated the mean and standard deviation of the scores and provided feedback to the team. In the second round, members adjusted their ratings based on the statistical results and feedback from the first round until the scores stabilized and consensus was reached. Two rounds of scoring were conducted in total, with the project leader summarizing and feeding back the results after each round.

To enhance methodological transparency and minimize potential subjectivity in scoring, several control measures were implemented: (i) prior to scoring, all team members received standardized training, with clear definitions of scoring dimensions and levels provided in a rating manual; (ii) an anonymous scoring procedure was adopted, and only aggregate statistics rather than individual scores were disclosed during the feedback stage; (iii) items with large discrepancies were discussed collectively and rescored to reach agreement; and (iv) to ensure consistency of the ratings, differences were progressively reduced through multiple Delphi rounds, feedback, and discussions until the scores stabilized, at which point consensus was considered to have been achieved.

Finally, the Risk Priority Number (RPN) for each failure mode was calculated as RPN = S × O × D, with a possible range of 1–1000. When the RPN value exceeds 125, corrective actions should be taken to address the issue (11). See Table 1.

**TABLE 1:** Risk values of Failure Mode and Effects Analysis before and after implementation.

Risk category	Potential failure modes	Pre-intervention RPN				Post-intervention RPN				Percentage decrease
		S	D	O	RPN	S	D	O	RPN	
Pre-Transdisciplinary Management	1. Incomplete Patient Assessment: Failure to accurately assess the patient’s condition and the suitability of cross-departmental treatment.	8.71	4.41	3.06	117.54	8.71	3.82	2.01	66.88	43.10%
	2. Insufficient Monitoring of Condition: Failure to timely detect and record changes in the patient’s condition before cross-departmental transfer, leading to incomplete information.	8.17	3.46	3.81	107.70	8.17	2.11	2.47	42.58	60.46%
	3. Inefficient Bed Resource Allocation: Bed allocation did not adequately consider the patient’s condition, urgency, and cross-departmental needs.	9.11	5.43	3.37	166.70	9.11	2.03	2.13	39.39	76.37%
	4. Ineffective Communication: Inefficient sharing of patient information between departments, leading to delays or omissions of key information.	8.15	4.23	3.06	105.49	8.15	2.25	2.08	38.14	63.84%
	5. Insufficient Integration of Cross-Departmental Resources: Lack of coordination between departments regarding equipment, personnel, etc., impacting patient transfer and subsequent treatment.	9.19	5.38	3.35	165.63	9.19	2.32	2.43	51.81	68.72%
Intra-Transdisciplinary Management	6. Inadequate Transfer of Materials: Transfer materials were not clearly labeled or the handover records were incomplete, affecting subsequent treatment of the patient.	8.81	4.32	2.92	111.13	8.81	3.16	2.05	57.07	48.65%
	7. Non-Standardized Patient Management: Inadequate patient safety monitoring mechanisms during cross-departmental transfers, leading to potential omissions or risks.	9.01	3.52	4.18	132.57	9.01	1.98	3.01	53.70	59.49%
	8. Insufficient Dynamic Monitoring: Nurses in the receiving department failed to accurately monitor the patient’s vital signs or condition changes.	9.88	4.13	4.51	184.03	9.88	1.28	2.85	36.04	80.42%
	9. Delayed Communication of Information: During the cross-departmental transfer, relevant healthcare personnel failed to promptly communicate the patient’s status and needs.	8.02	2.97	4.61	109.81	8.02	1.76	3.16	44.60	59.38%
	10. Delayed Specialist Response: Relevant specialty teams did not provide timely support, leading to delayed patient treatment or adjustment of the care plan.	9.21	3.10	4.05	115.63	9.21	2.04	2.34	43.96	61.98%
Post-Transdisciplinary Management	11. Unclear Handover when Returning to Original Department: After returning to the original department, nursing records and treatment plans were incomplete or unclear.	7.12	3.81	3.51	95.22	7.12	2.17	2.55	39.40	58.62%
	12. Lack of Continuity in Cross-Departmental Nursing: Departments did not collaborate in formulating subsequent nursing plans, leading to discontinuity in the patient’s rehabilitation process.	9.04	4.61	3.09	128.77	9.04	1.87	2.54	42.94	66.65%
	13. Insufficient Patient Safety Monitoring: Post-cross-departmental safety risks were not promptly identified, such as medication, treatment, or nursing-related risks.	9.59	3.33	3.42	109.22	9.59	2.47	2.78	65.85	39.71%
	14. Lack of Patient Feedback Mechanism: No long-term feedback system was established for cross-departmental treatment, affecting service improvement.	8.12	4.11	3.09	103.12	8.12	1.69	2.38	32.66	68.33%
	15. Inadequate Health Education Implementation: Failure to timely provide patients with comprehensive disease knowledge and education regarding cross-departmental treatment.	8.31	5.12	3.51	149.34	8.31	2.38	2.46	48.65	67.42%

### 2.3 Improvement measures

#### 2.3.1 Hospital-level improvement measures

Under the hospital-wide “One Bed” model, our hospital established an organizational framework led by the hospital president, with collaboration from the Medical Affairs Department, Pharmacy Department, Operations Department, Information Technology Department, Nursing Department, Finance Department, and Logistics Support Department. This multidisciplinary structure aimed to enhance team coordination and develop relevant management protocols. A regular cross-departmental communication mechanism was established, with multidisciplinary team meetings held at least once a week. The directors and head nurses of the top five departments with the highest cross-departmental patient admissions and discharges were required to attend these meetings. Under the guidance of hospital leadership and department directors, the team conducted repeated investigations to identify key challenges and bottlenecks in cross-departmental patient admissions. Multiple approaches, including in-depth interviews and structured questionnaires, were employed to pinpoint the underlying issues.

To optimize the cross-departmental admission principles, the hospital introduced a set of refined guidelines, including but not limited to: prioritizing specialty similarity, proximity of floor location, and allocating beds based on a “near-first, far-later” principle. Additionally, certain specialized departments were exempt from the unified bed allocation to ensure the availability of specialized care. Patients were promptly transferred back to their original specialty department as soon as a bed became available.

However, patient transfers may introduce additional challenges and risks. Studies have shown that intra-hospital transfers are associated with an increased risk of influenza infections (12). Each patient transfer not only increases the workload of nursing and cleaning staff but also reduces the time nurses can dedicate to direct patient care, potentially impacting the quality of nursing services.

In response, our hospital strengthened risk assessment and management during cross-departmental admission and patient transfer. Specifically, the Nursing Department coordinated the development of five standardized nursing consensus guidelines for surgical patients to support nurses involved in cross-departmental care. These guidelines included preoperative preparation checklists for surgical specialties, a unified protocol for surgical skin preparation, standardized management of surgical drainage tubes, a perioperative enhanced recovery after surgery (ERAS) nursing consensus, and disease-specific standardized postoperative health education protocols.

All consensus documents were shared across departments through an integrated information platform, allowing nurses to access relevant guidance in real time and facilitating the timely identification and management of key challenges and potential risks in cross-departmental patient care. In addition, the Nursing Department developed standardized health education videos covering disease-related knowledge as well as preoperative, intraoperative, and postoperative nursing care. These materials were delivered via electronic bedside display systems in inpatient ward to provide patients with consistent and structured health education throughout hospitalization.

Together, these measures supported patient safety during cross-departmental transfers, optimized the allocation of nursing resources, and reduced potential risks associated with patient movement. Furthermore, the hospital implemented a bidirectional incentive system for cross-departmental nursing performance, incorporating the number of cross-departmental patients, case complexity, and other relevant indicators to more accurately reflect nursing workload and its value. This approach contributed to the ongoing optimization of hospital-wide bed management.

#### 2.3.2 Department-level improvement measures

Under the hospital-wide “One Bed” model, each department may serve as both a receiving and discharging department for cross-departmental patients. Effective patient flow management, guided by the primary goal of patient safety, relies on clinical judgment, experience, and the expertise of healthcare professionals (13). To ensure the safe and effective implementation of nursing care under the “One Bed” model, enhancing the professional skills and overall competencies of nursing staff is essential.

Cross-departmental patient admission challenges traditional discipline-based nursing roles and places higher demands on nurses’ core competencies. Existing nurse competency development systems are insufficient to meet the workforce requirements of the hospital-wide “One Bed” model. Therefore, the nurse training program was designed based on the Iceberg Model and the Core Competencies Framework for Registered Nurses in China. Guided by these frameworks, a competency-oriented training curriculum aimed at breaking specialty boundaries was developed, encompassing eight core domains: clinical nursing practice, critical thinking, patient education and consultation, management and coordination, research and innovation, professional values and ethical–legal practice, professional development, and interpersonal relationship maintenance.

The development of generalist nurses has become a key measure to support hospital reform and promote the smooth implementation of the “One Bed” management model. The Nursing Department will lead and implement a series of professional training programs designed to enhance nurses’ knowledge and skills across multiple disease areas. The training covered, but was not limited to, theoretical knowledge of high-frequency cross-departmental diseases, clinical operation skills, emergency rescue techniques, standardized nursing documentation practices, key points for patient condition monitoring, and effective communication skills. In addition, the curriculum included modules on potential risk identification for high-risk and perioperative patients, integrated analysis of clinical information and individualized care planning, utilization of multidisciplinary resources, application of diversified patient education tools, emergency nursing workforce allocation, literature retrieval, professional resilience and stress management, as well as specialty-related ethical and legal issues.

To ensure the effectiveness of the training, a comprehensive assessment mechanism will be established. The training program will be available to all nurses, with particular attention given to nurses in the top five departments most frequently involved in cross-departmental patient care, as well as junior nurses. A blended learning approach, combining both online and offline methods, will be adopted to accommodate different learning preferences and scheduling needs. The training will be conducted weekly to ensure continuous knowledge updates and ongoing skill enhancement.

Additionally, departments will optimize shift scheduling by pairing senior and junior nurses to ensure nursing quality and safety. For cross-departmental patients, the receiving department will be required to provide health education at key time points, including upon admission, daily during hospitalization, and on the day of discharge or transfer. When the patient is transferred back to their original specialty department, health education will again be provided daily and on the day of discharge. The head nurse of each department will oversee the quality control of health education to prevent omissions.

To address vulnerabilities during night shifts, mobile nursing staff will be assigned to assist with the preparation of supplies and the environment for cross-departmental patient admissions. This measure aims to reduce the workload of on-duty nurses, thereby maintaining nursing service quality and ensuring patient safety.

#### 2.3.3 Logistics and support improvement measures

The successful implementation of the hospital-wide “One Bed” model relies heavily on the support of information technology (IT) to ensure smooth processes and patient safety. Our hospital’s information system is designed based on clinical needs, with regular assessments conducted at least once a week in the top five departments with the highest number of cross-departmental patient admissions and discharges. These visits allow the IT team to promptly identify and address any issues or inconveniences encountered by the clinical information system. This ensures the timely and accurate transmission of key patient information–such as medical records, treatment outcomes, and nursing needs–across departments. By preventing information silos, this approach reduces the risk of medical errors and enhances care coordination.

Due to the high cost of medical equipment procurement, the hospital cannot fully equip all receiving departments with backup devices. To address this limitation, the hospital has optimized centralized equipment management. This involves clearly defining the types, quantities, and utilization status of medical equipment, allowing for flexible interdepartmental deployment and resource sharing. This initiative enhances the availability and accessibility of essential equipment, supporting the continuity of care for cross-departmental patients.

**FIGURE 2:**
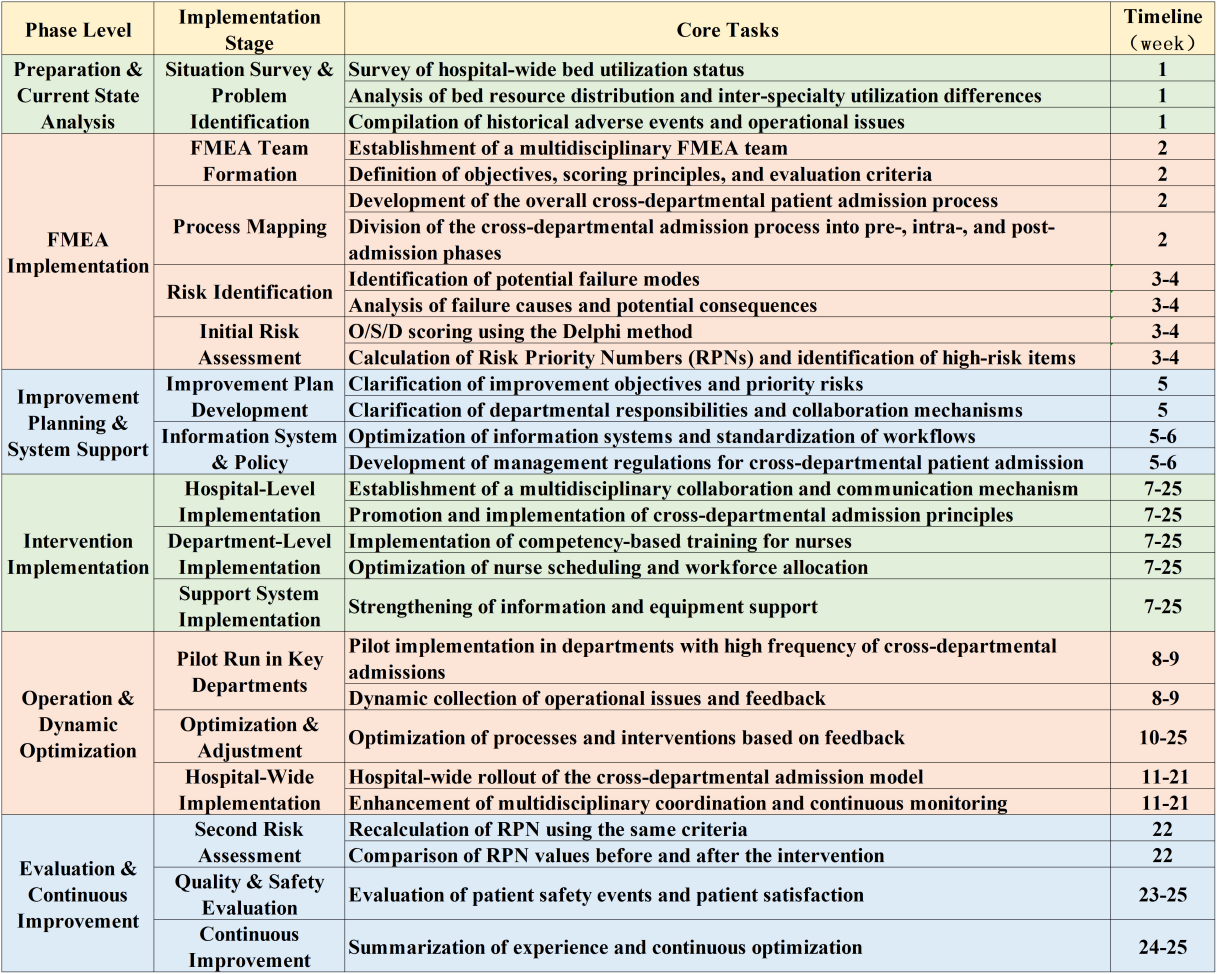
Failure Mode and Effects Analysis (FMEA)-based implementation phases and key activities.

The detailed implementation phases and corresponding activities of this study are illustrated in Figure 2.

## 3 Results

A total of 728 cross-departmental patients were admitted across all specialties in the hospital from January to June 2024. Among them, 530 patients (72.80%) were admitted through the emergency department, and 373 patients (51.24%) were admitted during nighttime hours (17:30–08:00). The top three specialties with the highest number of discharges to other departments were general surgery, neurosurgery, and gastroenterology.

The primary admission diagnoses for cross-departmental patients included abdominal pain, acute appendicitis, cerebrovascular accident, gastrointestinal bleeding, and acute pancreatitis, along with cases involving multiple trauma from falls, open craniocerebral injuries, and aortic dissection. On average, cross-departmental patients were transferred back to their original specialty department 1.3 days after admission.

**FIGURE 3:**
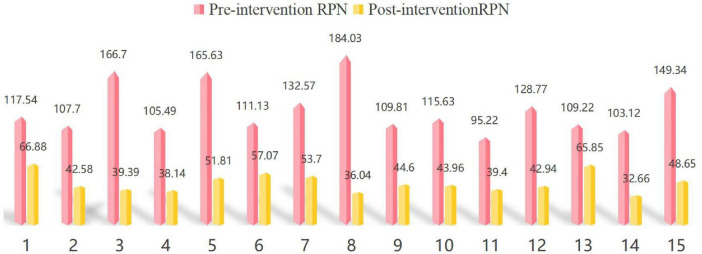
Pre- and post-intervention RPNs for key failure modes under the hospital-wide “One Bed” model.

Following the implementation of the improvement measures, the Risk Priority Numbers (RPNs) of the identified failure modes were recalculated. A comparison of the RPN values before and after the FMEA-based nursing quality improvement intervention under the hospital-wide “One Bed” model (Figure 3 and Table 1) showed that all RPN values decreased, with reductions ranging from 39.71% to 80.42%, indicating a substantial reduction in nursing quality risks across the cross-departmental management phases.

In addition, following the FMEA-based intervention, patient safety and nursing quality indicators showed improvements. The incidence of in-hospital pressure injuries decreased from 0.034% to 0.024%, falls from 0.128‰ to 0.096‰, unplanned extubations from 0.26% to 0.19%, and central line-associated bloodstream infections from 0.027‰ to 0.017‰. Patient satisfaction increased from 93.32 to 95.88 (on a 100-point scale), and the average preoperative waiting time decreased from 6.99 days to 2.96 days.

## 4 Discussion

### 4.1 Patient flow management under the “One Bed” model

Effective patient flow management requires the identification of patients, nursing processes, process managers, and frontline staff. It has a broad impact on patient outcomes, system efficiency, and staff performance (14). The hospital-wide “One Bed” model integrates in-hospital resources to meet patients’ timely admission needs, effectively alleviating the issue of “difficulty in hospital admission.”

Currently, with policy support from multiple regions in China, the “One Bed” model is being continuously piloted and refined (15). In March 2024, the Beijing Municipal Health Commission issued the 2024 Beijing Medical Service Improvement Plan, mandating tertiary hospitals to expand day medical services and pilot the “One Bed” management model. In May 2023, China’s National Health Commission released the Notice on Improving the Patient Experience, encouraging hospitals to centrally manage idle beds and gradually implement hospital-wide centralized bed allocation and scheduling. Similarly, in July 2023, the Shanghai Municipal Health Commission, together with other departments, issued the Shanghai Action Plan for Improving the Patient Experience, promoting the centralized management of vacant beds to enhance resource utilization. In June 2024, the Sichuan Provincial Government released the Measures to Promote the High-Quality Development of Public Hospitals in Sichuan Province, outlining initiatives such as standardizing hospital branch settings and bed scale management to ensure the rational allocation and efficient utilization of medical resources.

### 4.2 Enhanced risk management through FMEA

Against this backdrop, the present study employed a systematic risk identification and analysis process to reveal potential vulnerabilities in nursing quality management. Specifically, the FMEA methodology enabled the quantification of the Risk Priority Numbers (RPNs) for each failure mode, facilitating cross-departmental collaboration and the integration of multidisciplinary insights to identify key areas for improvement.

Following the implementation of the corrective measures, the RPN values of failure modes significantly decreased, indicating that the interventions effectively enhanced nursing quality. The core strength of nursing quality management lies in its patient-centered, comprehensive nursing model. The “One Bed” model ensures the systematic and continuous delivery of medical and nursing care by optimizing nursing plans, improving cross-departmental handovers, and promoting multidisciplinary nursing consultations. These measures collectively enhance the consistency and quality of nursing services (16, 17).

However, the current nursing management processes under the “One Bed” model remain incomplete. Successful implementation requires multidisciplinary collaboration across departments, including logistics support, IT system refinement, rational allocation of ward resources, cross-departmental communication, and continuous monitoring of healthcare quality (18, 19). Although some hospitals have achieved initial success during the pilot phase, the scalable implementation of this model across hospitals of different regions and levels requires further exploration. Additionally, the sustainability of nursing management processes under the “One Bed” model needs to be validated through ongoing practice and evaluation.

### 4.3 Future prospects: leveraging digital and intelligent technologies

In the era of digital healthcare, the application of intelligent technologies is expected to further enhance the quality and efficiency of nursing services under the “One Bed” model. The integration of IT and mobile internet technologies has already provided convenient access to various aspects of medical and nursing processes (20).

For instance, innovations such as contactless smart healthcare platforms, WeChat-based medical service platforms, integrated facial recognition systems for insurance services, mobile healthcare service apps, and cloud-based healthcare management systems significantly enrich the patient experience and enhance the efficiency and accuracy of medical and nursing services (21–23).

Building on these experiences, such technologies could be further leveraged within the “One Bed” model to support FMEA processes, including risk identification, RPN assessment, and tracking of improvement measures. Mobile apps and WeChat platforms could facilitate task reminders and timely information sharing, while cloud-based systems and smart dashboards could optimize cross-department bed allocation and resource management, improving workflow efficiency and continuity of care.

In recent years, the Kanban method has gained attention for its application in hospital bed management. To optimize emergency bed management, Benjamin et al. (24) developed the Cuidartech Kronos software, which provides reliable data support for healthcare professionals and administrators, thereby improving workflow efficiency and enhancing patient care quality. Furthermore, electronic Kanban tools combined with network access by the bed management team have been shown to effectively reduce patient length of stay (25). Building on this, Grüble et al. (26) applied a hybrid system that combines artificial neural networks (ANNs) with multi-branch value theory to enhance contextual awareness in bed management. By automating the bed allocation process, their model achieved a 93.5% similarity between the system’s bed assignments and those made by hospital administrators, demonstrating its high accuracy in bed matching.

As a solid technological foundation for nursing services, information technology plays a crucial role in optimizing workflows, improving efficiency, and promoting precision nursing and medical care. The integration of intelligent technologies within the “One Bed” model is expected to significantly enhance the overall quality of healthcare services and patient experiences, while also supporting structured FMEA-based quality improvement initiatives.

## 5 Limitations

This study has several limitations. First, it was conducted at a single center over a relatively short period, with a limited sample size, which may constrain the generalizability of the findings. Future studies involving multi-center designs and larger sample sizes are warranted to validate the applicability of the intervention across diverse hospital settings. Second, the long-term effects and sustainability of the implemented improvement measures have not been evaluated, and their enduring impact should be examined in subsequent studies.

Additionally, the FMEA process relies on expert judgment for risk assessment, which inherently introduces a degree of subjectivity that may influence the calculated risk priority numbers (RPNs). In the present study, all team members received comprehensive training, scoring criteria were standardized, and scoring was conducted anonymously to mitigate subjectivity. For future applications, additional strategies could be adopted, such as integrating objective clinical data (e.g., patient safety events, adverse incident reports) and implementing blinded scoring procedures where feasible. These measures may enhance the reliability, reproducibility, and overall rigor of FMEA-based risk assessments.

## 6 Conclusion

This study systematically analyzed nursing quality improvement under the hospital-wide “One Bed” model using the FMEA approach. The results indicate that this model can effectively identify potential risks, optimize nursing processes, and enhance nursing quality while ensuring patient safety. It should be noted that the FMEA process relies on expert judgment, which may introduce a degree of uncertainty; therefore, the results should be interpreted with caution. Future studies could further explore strategies to mitigate uncertainty and validate the applicability of this approach across different hospital settings.

## 7 Relevance to clinical practice

This study provides a structured reference for hospitals implementing the hospital-wide “One Bed” model. By applying FMEA to identify high-risk points in cross-departmental nursing processes, nursing teams can proactively manage potential failures, optimize care pathways, and enhance patient safety. The multidisciplinary collaboration framework and targeted nurse training described in this study offer practical guidance for improving workflow coordination and resource allocation. In addition, the use of information systems for systematic monitoring is proposed as a potential approach to support timely communication of patient information and improve the efficiency of bed management. Collectively, these findings can assist nursing managers and healthcare administrators in developing evidence-based strategies to enhance care quality, reduce safety risks, and promote more effective utilization of hospital-wide bed resources.

Furthermore, this study provides multiple direct benefits for nursing staff. Nurses are able to better recognize potential failure modes and associated risk factors in patient care, thereby enhancing vigilance and proactive risk management. The identification of high-priority risks and corresponding corrective measures offers practical, evidence-based guidance to optimize workflows and reduce errors. Moreover, cross-departmental coordination and multidisciplinary discussions strengthen nurses’ communication skills and teamwork. Participation in FMEA scoring and quality improvement initiatives also supports the development of clinical judgment, decision-making skills, and nursing quality management capabilities.
